# Load-bearing mechanism and engineering application of a heavy-duty transfer steel platform supported by basement columns

**DOI:** 10.1371/journal.pone.0336277

**Published:** 2025-12-08

**Authors:** Meijun Shang, Xuemei Li

**Affiliations:** School of Visual Arts, Changchun Sci-Tech University, Changchun, Jilin, China; Tongji University, CHINA

## Abstract

Heavy lifting operations performed on basement roof slabs often impose concentrated loads that may cause local stress concentrations, cracking, or even structural failure. To address this issue, this study proposes a heavy-load transfer steel platform supported by basement columns, which effectively isolates the lifting load from the roof slab to ensure structural safety. The load-bearing mechanism of the proposed steel platform was analyzed theoretically, and finite element analysis (FEA) was employed to evaluate the stresses and deflections of key members. A particle swarm optimization (PSO) algorithm was integrated with the FEA model to optimize the cross-sectional dimensions of the primary beams, secondary beams, and foundation boxes, achieving a balance between load-bearing capacity and cost efficiency. The method was validated through field measurements from the Phase I project of the Hangzhou Convention and Exhibition Center, where strain gauges and displacement sensors were installed at critical positions for real-time monitoring. The measured data showed good agreement with the FEA predictions, with deviations of 5.2% for steel beam stress and 3.7% for foundation box deflection. After optimization, the material usage of the foundation boxes, secondary beams, and primary beams was reduced by 44.68%, 58.33%, and 55.00%, respectively, resulting in an overall material cost reduction of 52.67%. The results demonstrate that the proposed platform effectively mitigates stress concentration and prevents cracking of basement roof slabs under large-tonnage hoisting conditions. The structure exhibits high safety, efficiency, and reusability. Furthermore, the use of recyclable steel materials aligns with green construction and sustainability principles. Future research should explore the platform’s applicability under irregular column layouts and dynamic loading conditions.

## 1. Introduction

With the continuous acceleration of modern urbanization and the rapid development of the construction industry, the demand for underground structures has been increasing, particularly in large-scale commercial projects, exhibition halls, and other venues. As a critical component of buildings, basements are widely used in various construction projects. However, with the popularization of large lifting machinery, the issue of the load-bearing capacity of basement roof slabs during the construction of superstructures has become increasingly prominent. Basement roof slabs are typically designed to bear relatively light loads, but the increase in heavy lifting machinery and other equipment has significantly raised the pressure exerted by lifting loads on the roof slab, which can lead to cracking or severe structural damage [1]. Traditional reinforcement methods, especially scaffold-supported reinforcement, face several problems, including uneven load transfer, long construction cycles, and high project costs. Moreover, when subjected to large loads, local overloads in scaffold supports may cause the basement roof slab to crack [2,3].

Against this backdrop, it is essential to explore innovative methods capable of effectively addressing the load-bearing issues of basement roof slabs. To efficiently transfer hoisting loads to the underground structure while minimizing damage to the structure and ensuring construction safety, researchers have carried out extensive studies on related aspects, including structural reinforcement and load transfer, slab stress mechanisms, material and structural performance, and construction technology and optimization design.

In terms of structural reinforcement and load transfer, Shentu et al. (1997) conducted a detailed analysis of the load-bearing capacity of concrete slabs and proposed a theoretical model for structural reinforcement [4]. Ospina and Alexander (1998) focused on studying the load transfer mechanism of concrete columns through slabs, providing a theoretical basis for optimizing the load transfer system [5]. Alani et al. (2014) investigated the structural behavior and deformation patterns of ordinary concrete floor slabs under load and suggested recommendations for strengthening designs [6]. Teng and Zhang (2015) used nonlinear finite element methods to analyze the behavior of fiber-reinforced polymer (FRP)-reinforced concrete slabs under cyclic loads at fixed points [7]. Henze et al. (2020) proposed a new shear design method based on experimental data to enhance the load-bearing capacity of reinforced concrete slabs under concentrated loads [8]. De Sousa et al. (2023) analyzed the failure mechanism of unidirectional slabs after partial reinforcement under concentrated loads, further validating the effectiveness of the reinforcement design [9]. Zhou et al. (2024) studied reinforcement designs for slab openings in subway stations, proposing multiple reinforcement methods and analyzing their impact on slab load-bearing capacity [10]. Chen et al. developed a modular suspended sliding scaffold, which significantly enhanced construction efficiency and safety [11].

Regarding slab stress mechanisms, Abdel-Sayed et al. (1974) studied the dynamic load response of composite slabs, providing theoretical support for the design of composite structures [12]. Boothby and Laman (1999) examined the damage caused by vehicle loads on bridge deck concrete slabs and proposed related fatigue analysis models [13]. Maekawa et al. (2006) explored the fatigue behavior of reinforced concrete slabs under traffic wheel loads and proposed a three-dimensional fatigue simulation method [14]. Meng et al. (2019) employed finite element analysis to study the long-term deformation prediction of pre-stressed concrete bridges under heavy traffic loads, proposing optimized design and prediction schemes [15]. Gao et al. (2020) studied the fatigue performance of reinforced concrete bridge deck slabs under vehicle loads, emphasizing the impact of steel plate usage on structural durability [16]. Hung et al. (2020) further explored the high-frequency fatigue interactions between soil foundations and concrete slabs, revealing structural responses under moving loads through finite element analysis [17]. Cajka et al. (2020) conducted numerical modeling to analyze the impact of soil-structure interactions on concrete slabs, highlighting the effect of different soil properties on slab performance [18]. Duris and Hrubesova (2020) studied the interaction between fiber concrete slabs and soil, discussing the main factors influencing this process [19]. Xie and Wang (2023) compared methods to mitigate cracks in airport composite pavements, proposing pavement structure modifications to reduce reflection cracking [20]. Wang et al. (2023) performed multi-fractal analysis on fatigue cracks in reinforced concrete hollow core beams, revealing crack propagation patterns and providing references for structural health monitoring [21]. Jagadeesh et al. (2025) simulated the movement of forklifts on concrete pavements using finite element models, studying load distribution caused by tire-ground contact [22]. Shaikhjan and Jha proposed the “contact stiffness method”, which calculates the load distribution between the floor slabs and the supporting system during the construction phase through the stiffness matrix, providing a more accurate reflection of the stress characteristics of concrete slabs at different curing ages [23].

In terms of material and structural performance, Taylor et al. (1966) explored the impact of steel reinforcement layout on the behavior of concrete slabs, proposing how to reasonably configure reinforcement to improve structural performance [24]. Sorelli et al. (2006) studied the application of steel fiber reinforced concrete in ground slabs, finding that steel fibers effectively enhance the cracking resistance and load-bearing capacity of concrete [25]. Rizzuto et al. (2022) conducted large-scale loading experiments to study the structural behavior and theoretical analysis of steel mesh-reinforced concrete floor slabs, providing important experimental data for slab design [26]. Liu et al. (2024) used improved GPR and FSF methods to predict deflections of railway-road suspension bridges under multiple operational loads, providing important references for bridge design [27]. Peng et al. identified the main failure mechanisms of the concrete formwork support system and proposed an improvement measure to enhance overall stability by strengthening the diagonal bracing [28].

In terms of construction technology and optimization design, Taylor and Hayes (1965) experimentally tested the impact of edge constraints on the punching shear behavior of reinforced concrete slabs, providing valuable experience for slab design [29]. Hansen et al. (2002) studied the mechanism of transverse cracking in concrete pavements and provided a detailed crack analysis model for road engineering design [30]. Nassif et al. (2009) investigated the field performance of bridge approach slabs and evaluated the impact of different construction methods on structural safety [31]. Alrousan and Bara’a (2022) studied the punching shear capacity of concrete slabs under different opening configurations and load conditions, offering new theoretical support for concrete structure design [32]. Kim et al. found that the semi-rigidity of joints in the prefabricated steel bracing system significantly reduces the overall stiffness. Proper bracing arrangement can improve load-bearing capacity. They proposed an overall analysis method based on joint stiffness correction [33]. Setiawan et al. (2024) validated shear failure of bridge deck slabs under concentrated loads, providing an important basis for structural design [34]. Alqattan et al. used numerical analysis to reveal the stress characteristics and failure mechanisms of the wedge-shaped formwork joints. They proposed an optimal wedge angle design, which enhances the safety of the temporary support system [35].

The above studies offer valuable theoretical support and technical references for this research. However, most of the studies focus on ground slabs, beams, and concrete reinforcement, with relatively few investigations into the load distribution of basement roof slabs and hoisting load transfer systems. Especially when heavy lifting machinery directly exerts loads on the basement roof slab, there is a lack of systematic research and application cases on how to effectively prevent slab cracking through reasonable stress system designs. This paper proposes a new heavy-duty transfer steel platform and corresponding design methods. This steel platform effectively avoids the load concentration problems of traditional scaffold reinforcement methods. By transferring loads to the basement columns and eventually to the foundation through piles, it prevents excessive pressure on the basement roof slab. This research systematically analyzes the load-bearing mechanism of the heavy-duty transfer steel platform supported by basement columns and proposes corresponding design optimization methods, providing new ideas and methods for solving underground structure reinforcement issues.

## 2. Design concept of the heavy-load transfer steel platform

In modern construction projects, particularly in the erection of large-span steel structures, the use of heavy lifting machinery has become increasingly widespread. Such equipment is often required to operate on basement roof slabs. In large-scale projects such as exhibition centers and museums, the loads imposed by cranes can subject the basement roof slabs to enormous pressure. If this pressure is not effectively dispersed and transmitted, it may easily cause cracking or even structural damage. Conventional reinforcement methods, such as scaffolding supports, can mitigate this problem to a certain extent; however, they present several drawbacks, including concentrated load transfer, extended construction periods, high costs, and significant safety risks. Therefore, it is imperative to develop an innovative reinforcement method that not only effectively disperses loads but also prevents excessive stress on the basement roof slabs.

### 2.1. Traditional scaffolding support reinforcement

**Fig 1:**
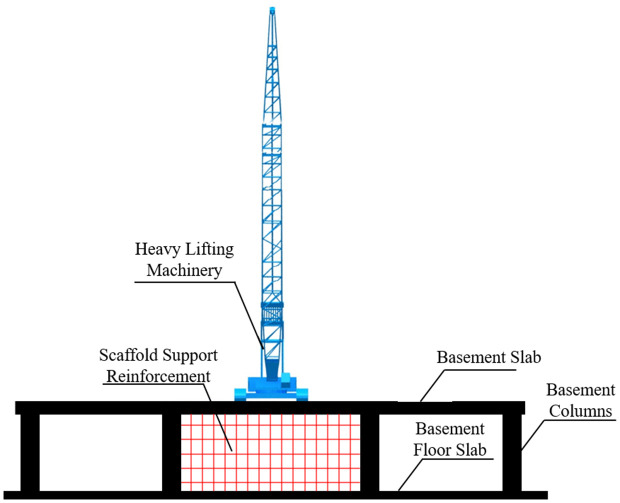
Traditional scaffolding reinforcement system.

As illustrated in Fig 1, the conventional reinforcement method involves the installation of a series of scaffolding structures beneath the basement roof slab to help distribute heavy loads. Typically, this method employs vertical support poles and horizontal beams, which rely on their stiffness and strength to share the applied loads, thereby preventing the roof slab from cracking under excessive concentrated stresses.

However, the practical application of scaffolding reinforcement reveals a number of shortcomings. The foremost issue is the poor performance in load dispersion. While the scaffolding system can transmit loads to the basement roof slab, the distribution remains concentrated in localized areas, which easily induces stress concentrations. These concentrated stresses may lead to cracking or localized structural damage of the slab. Furthermore, scaffolding reinforcement systems demand a considerable number of support members, particularly for large-span or heavy-lift operations, occupying significant space and increasing construction height. This not only complicates construction procedures but also prolongs the overall schedule.

In projects requiring high strength and precision, the stiffness and stability of scaffolding may be inadequate to withstand sudden load variations or extreme environmental conditions, thereby jeopardizing construction safety. In addition, the extensive occupation of construction space by scaffolding systems introduces further safety risks. Due to the unreliability of load distribution, uneven stress conditions can cause deformation, slippage, or even collapse of the scaffolding, posing hazards to both personnel and equipment.

Another limitation is the high construction and maintenance costs. Although the initial investment in scaffolding structures may appear relatively low, the large number of components and complex installation procedures significantly increase total costs. Moreover, scaffolding systems require regular inspection and maintenance to prevent damage or deformation from prolonged use, thereby adding further complexity and expense to the construction process.

### 2.2. Heavy-load transfer steel platform

**Fig 2:**
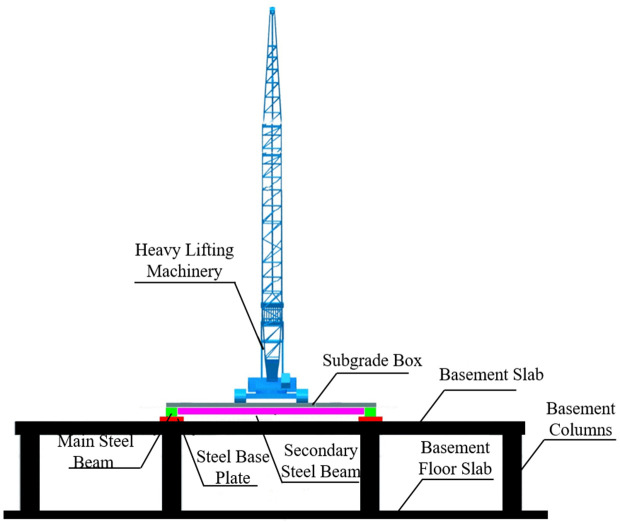
Heavy-load transfer steel platform.

To address the deficiencies of scaffolding reinforcement, a novel heavy-load transfer steel platform is proposed, as illustrated in Fig 2. The platform is primarily composed of foundation boxes, secondary steel beams, primary steel beams, and steel base plates. Importantly, a clearance is maintained between the foundation boxes and the basement roof slab. This ensures that even under maximum deformation, the foundation boxes do not contact the slab, thereby preventing the roof slab from bearing heavy loads.

### 2.3. Comparison of load transfer paths between the two modes

**Fig 3:**
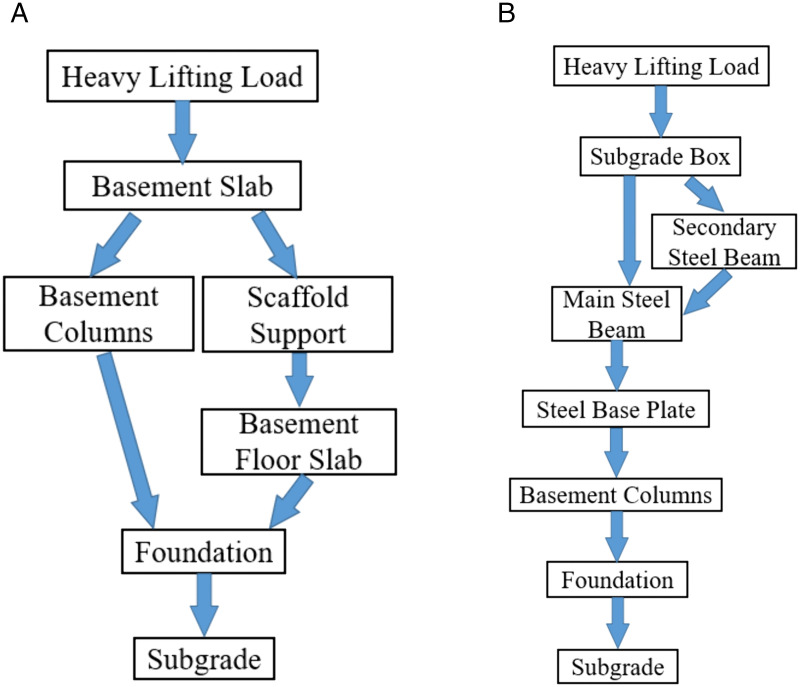
Comparison of load transfer modes: (a) Traditional scaffolding reinforcement; (b) Heavy-load transfer steel platform.

Under heavy lifting loads, the load transfer mechanisms of the traditional scaffolding reinforcement system and the heavy-load transfer steel platform differ significantly, as shown in Fig 3(a) and Fig 3(b). In the traditional scaffolding reinforcement mode, the load from lifting equipment acts directly on the basement roof slab. Through the scaffolding system set up beneath the slab, the load is gradually transferred to the foundation. The detailed load transfer process is as follows:

(1) Heavy lifting load: The load is applied to the basement roof slab, concentrated at the crane contact points, which may cause localized overstressing and cracking.
(2) Basement roof slab: The slab transmits the concentrated load to the scaffolding system beneath, potentially leading to excessive localized stresses and structural damage.
(3) Scaffolding system: The load is then transferred to the basement floor through vertical poles and horizontal beams.
(4) Basement columns and foundation: A portion of the load is borne by the basement columns and eventually dispersed into the foundation soil.

In contrast, the heavy-load transfer steel platform mode employs a specially designed steel platform to achieve more uniform load dispersion, preventing the roof slab from participating in load transfer. The process is as follows:

(1) Heavy lifting load: The load is first borne by the foundation boxes and then transmitted through the primary and secondary steel beams to the basement columns. Since the clearance between the foundation boxes and the roof slab exceeds the maximum possible deflection of the boxes, the slab remains non-load-bearing.
(2) Steel platform: The structural configuration of the platform ensures even load distribution, eliminating concentrated stress on the basement roof slab. All loads are transferred through the platform supports to the basement columns.
(3) Basement columns and foundation: The platform is connected to the columns through steel base plates, transferring loads safely into the foundation and ultimately dispersing them into the subgrade. Compared to the conventional approach, this system achieves significantly more uniform load transfer, avoiding localized overstressing.

Table 1 highlights the key differences between the two reinforcement modes.

**Table 1:** Comparison between heavy-load transfer steel platform and traditional scaffolding reinforcement.

Item	Traditional scaffolding reinforcement	Heavy-load transfer steel platform
Load dispersion	Concentrated, prone to local stress concentration	Uniform dispersion, reduced local stress
Roof slab capacity	Limited, prone to cracking	Roof slab not engaged, no risk of cracking
Construction safety	Susceptible to instability, higher risks	Stable structure, high safety
Efficiency & cost	Long cycle, high cost	Short cycle, cost-effective
Adaptability	Suitable only for simple lifting	Highly adaptable to various operations

(1) Load dispersion: In the scaffolding mode, crane loads are directly applied to the basement roof slab, producing local stress concentrations and possible cracking. In the steel platform mode, loads are dispersed over a larger area, effectively reducing localized stresses.
(2) Roof slab bearing capacity: In the scaffolding mode, concentrated loads limit the slab’s bearing capacity and may cause failure under heavy loads. In the steel platform mode, the slab is completely relieved of load, avoiding any risk of damage.
(3) Construction safety: Scaffolding systems are prone to instability under sudden load variations, whereas the steel platform provides greater stiffness and stability, ensuring safer construction.
(4) Efficiency and cost: Scaffolding systems require extensive onsite assembly, leading to longer construction periods and higher material costs. Steel platforms, by contrast, allow for prefabrication, rapid assembly, and reusability, thus improving economic efficiency.
(5) Adaptability: While scaffolding reinforcement is limited to simple lifting operations, the steel platform is versatile and suitable for large-span and heavy lifting applications, including high-rise and underground projects.

Through comparative analysis, it is evident that the heavy-load transfer steel platform offers significant advantages in mitigating the risk of basement roof slab cracking under heavy lifting loads. By effectively dispersing loads and bypassing the roof slab, the platform enhances both safety and efficiency. Compared with traditional scaffolding reinforcement, it not only improves structural reliability but also reduces construction costs, making it particularly well-suited for large-span and heavy-duty lifting operations.

## 3. Structural analysis and optimization design method of the steel platform

### 3.1. Structural analysis of the steel platform

As illustrated in Figs 3, the heavy-load transfer steel platform represents an innovative reinforcement solution. Its fundamental concept lies in rationally transmitting loads from the basement roof slab to the underground structural columns, and ultimately to the foundation. This design breaks through the limitations of traditional scaffolding reinforcement methods, completely avoiding excessive local pressure on the basement roof slab induced by heavy lifting loads, thereby significantly reducing the risk of cracking.

The structural analysis of the steel platform is critical for determining the specifications and strength requirements of its components. From its structural composition, the platform can essentially be regarded as a beam–slab system. The analysis of its load-bearing behavior mainly involves three parts: (i) stress analysis of the foundation boxes, (ii) stress analysis of the primary and secondary steel beams, and (iii) stress analysis of the basement columns.

#### 3.1.1. Stress analysis of the foundation box.

The foundation box constitutes the slab component of the beam–slab system and directly resists the heavy lifting loads. It is primarily subjected to bending moments and shear forces under biaxial bending conditions. Its stress state should satisfy the following criteria [36]:


$${\left(\sigma_{xy}\right)}_{\max}={\left(\frac{M_{x}}{W_{nx}}+\frac{M_{y}}{W_{ny}}\right)}_{\max}\leq f$$ (1)

$$\tau_{\max}={\left(\frac{VS}{It_{w}}\right)}_{\max}\leq f_{v}$$ (2)

$${\left(\sqrt{\sigma^{2}+3\tau^{2}}\right)}_{\max}\leq f$$ (3)

Where, $\sigma_{xy}$ denotes the normal stress induced by biaxial bending; *M*_x_ and *M*_y_ are the bending moments about the principal axes *x* and *y*; *W*_nx_ and *W*_ny_ are the net section moduli about these axes; *f* represents the permissible flexural strength of the steel; τ denotes the shear stress; *V* is the shear force on the section; *S* is the first moment of area above the neutral axis; *I* is the gross moment of inertia; *t*_w_ is the section thickness; *f*_v_ is the permissible shear strength of the s*t*eel; and σ denotes the normal stress due to uniaxial bending. Equation (1) specifies the strength condition under biaxial bending, Equation (2) the strength condition under pure shear, and Equation (3) the condition under combined bending and shear.

Moreover, to ensure that the foundation box remains clear of the basement roof slab, its maximum deflection $w_{p\max}$ must always be less than the clearance $t_{p-f}$ between the bottom of the foundation box and the top surface of the roof slab, i.e.,:


$$w_{p\max}\leq t_{p-f}$$ (4)

#### 3.1.2. Stress analysis of primary and secondary steel beams.

Primary beams: The primary steel beams carry the majority of the load and are mainly subjected to bending moments and shear forces. Using finite element analysis (FEA), the maximum bending moment and shear force under the most unfavorable loading conditions can be determined.

Secondary beams: The secondary steel beams provide auxiliary support to the primary beams. Their main function is to share the load distribution and maintain platform stability. Their dimensions and layout must be consistent with the stress characteristics of the primary beams in order to avoid local overloading.

Specifically, both primary and secondary beams are classified as bending–shear members subjected to uniaxial bending. Their stress conditions must satisfy [37]:


$${\left(\sigma \right)}_{\max}={\left(\frac{M_{x}}{W_{nx}}\right)}_{\max}\leq f$$ (5)

In addition, they must also conform to the strength requirements of Equation (2) and Equation (3).

#### 3.1.3. Stress analysis of basement columns.

The basement columns sustain the axial forces and bending moments transmitted from the primary beams. Their strength requirements are governed by the following conditions [38]:


$${\left(\frac{N}{A_{n}}+\frac{M_{x}}{W_{nx}}\right)}_{\max}\leq f_{c}$$ (6)

$${\left(\frac{N}{A_{n}}-\frac{M_{x}}{W_{nx}}\right)}_{\max}\leq f_{c}$$ (7)

$${\left(\frac{N}{A_{n}}\right)}_{\max}\leq f_{c}$$ (8)

Where, *N* denotes the axial force carried by the column, and *f*_c_ is the permissible compressive strength of the column material.

#### 3.1.4. Deflection of beams and foundation box.

In addition to satisfying the condition shown in Equation (4) that ensures the foundation box and the basement roof slab remain in a non-contact state, the deflection of the beams and foundation box must be restricted to ensure the heavy load transfer steel platform can effectively serve its purpose in the construction process. Specifically, the maximum deflections are constrained as follows:


$$w_{p\max}\leq \left[w_{p\max}\right]$$ (9)

$$w_{mb\max}\leq \left[w_{mb\max}\right]$$ (10)

$$w_{sb\max}\leq \left[w_{sb\max}\right]$$ (11)

where $w_{mb\max}$ and $w_{sb\max}$ represent the maximum deflections of the primary steel beam and secondary steel beam, respectively, and $\left[w_{p\max}\right]$, $\left[w_{mb\max}\right]$, and $\left[w_{sb\max}\right]$ represent the allowable maximum deflections for the foundation box, primary steel beam, and secondary steel beam, respectively.

### 3.2. Intelligent optimization using particle swarm algorithm

#### 3.2.1. Principle.

The fundamental concept of the Particle Swarm Optimization (PSO) algorithm is to simulate the collective behavior of a group, where information sharing among individuals drives the evolution of the swarm from disorder to order in the solution space, ultimately yielding feasible solutions [39,40]. During the search process, each particle memorizes its historically optimal position, while sharing this information with other particles. Through such interactions, the swarm collectively identifies the most promising regions of the solution space, eventually converging to the global optimum [41].

The computational process of PSO involves iterative updates of velocity and position [42]. The velocity update is expressed as:


$$v_{i}^{k+1}=w\cdot v_{i}^{k}+c_{1}\cdot r_{1}\cdot (pbest_{i}-x_{i}^{k})+c_{2}\cdot r_{2}\cdot (gbest-x_{i}^{k})$$ (12)

Where, $v_{i}^{k}$ is the velocity of particle *i* at iteration *k*; $x_{i}^{k}$ is the position of particle *i* at iteration *k*; *pbest*_i_ denotes the individual historical best position; *gbest* is the global best position; *w* is the inertia weight controlling momentum; *c*_1_ and *c*_2_ are acceleration constants; and *r*_1_ and *r*_2_ are random numbers uniformly distributed in [0,1]. The position update is given as:


$$x_{i}^{k+1}=x_{i}^{k}+v_{i}^{k+1}$$ (13)

Where, $x_{i}^{k+1}$ is the updated position of particle *i* at iteration *k* + 1.

In the context of this study, PSO was selected due to its ability to effectively search high-dimensional solution spaces, ensuring an optimal design for the steel platform with respect to both load-bearing and cost efficiency. Compared to other optimization techniques such as genetic algorithms (GA) or gradient-based methods, PSO is more effective in handling complex, nonlinear objective functions with many variables. Furthermore, PSO provides a relatively straightforward implementation without requiring gradient information, making it suitable for problems where analytical solutions are not easily available or difficult to compute. Other algorithms, such as GA, were considered but found less efficient in converging to the optimal solution within the specific context of steel platform optimization, especially given the non-linear nature of the design parameters.

#### 3.2.2. Computational procedure.

**Fig 4:**
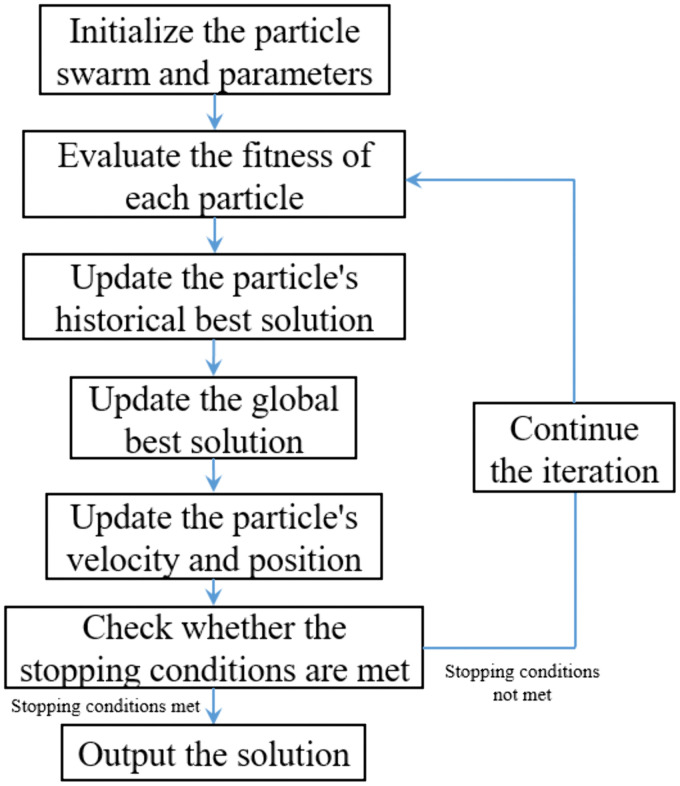
Computational Procedure of the Particle Swarm Optimization (PSO) algorithm.

As illustrated in Fig 4, the computational procedure of PSO consists of the following steps [43]:

(1) Initialization: Initialize particle positions and velocities; define parameters including inertia weight *w*, acceleration constants *c*_1_ and *c*_2_, and maximum iteration count; assign initial global best (*gbest*) and personal best (*pbest*_i_).
(2) Evaluation: For each particle, compute the objective function value, and update *pbest*_i_ and *gbest* accordingly.
(3) Velocity and position update: Apply Equation (9) and Equation (10) to update particle velocity and position.
(4) Termination check: Determine whether stopping criteria are met, such as maximum iteration count or predefined error tolerance.
(5) Output: Return the solution corresponding to *gbest* as the optimal solution.

In summary, PSO is a highly efficient global optimization method applicable to both continuous and discrete problems. By simulating swarm intelligence and information sharing, it can rapidly locate optimal solutions in high-dimensional search spaces.

### 3.3. Optimization design system of the steel platform

To ensure efficiency and safety in practical applications, optimization design of the steel platform is required. The objective is to configure structural components such that both load-bearing requirements and cost minimization are achieved.

#### 3.3.1. Optimization objective.

Let the total cost of the steel platform be $C_{SP}$, comprising the costs of the foundation boxes $C_{Rb}$, secondary beams $C_{SB}$, and primary beams $C_{MB}$. The optimization objective is to minimize cost:


$$\min C_{SP}=C_{Rb}+C_{SB}+C_{MB}=c_{Rb}m_{Rb}+c_{SB}m_{SB}+c_{MB}m_{MB}$$ (14)

where, $c_{Rb}$, $c_{SB}$, $c_{MB}$ denote the unit mass costs of the foundation box, secondary beam, and primary beam, respectively; and $m_{Rb}$, $m_{SB}$, $m_{MB}$ represent their corresponding masses. These masses are directly related to the optimization variables and can be expressed as:


$$m_{Rb}=\rho_{s}\left(\sum_{i=1}^{n_{Rb}}{l_{Rb}}_{i}A_{Rb}\right)$$ (15)

$$m_{SB}=\rho_{s}\left(\sum_{i=1}^{n_{SB}}{l_{SB}}_{i}A_{SB}\right)$$ (16)

$$m_{MB}=\rho_{s}\left(\sum_{i=1}^{n_{MB}}{l_{MB}}_{i}A_{MB}\right)$$ (17)

Where, $\rho_{s}$ is the density of steel; $n_{Rb}$, $n_{SB}$, $n_{MB}$ denote the number of foundation boxes, secondary beams, and primary beams; $l_{Rb}$, $l_{SB}$, $l_{MB}$ their respective lengths; and $A_{Rb}$, $A_{SB}$, $A_{MB}$ their cross-sectional areas. Thus, mass is obtained by multiplying cross-sectional area by length and density.

#### 3.3.2. Optimization parameters and constraints.

The optimization variables are the cross-sectional specifications of the foundation boxes, secondary beams, and primary beams. Their combinations directly influence both the internal forces and costs of the steel platform. Consequently, they must satisfy the strength conditions and deformation conditions prescribed in Equation (1)–Equation (11).

#### 3.3.3. Implementation of optimization.

In conclusion, the optimization objective corresponds to Equation (14), with optimization parameters being the cross-sectional specifications of the foundation boxes, secondary beams, and primary beams, while the constraints are given by Equation (1)–Equation (11). When parameters change, the cost can be recalculated via Equation (14)–Equation (17), and the corresponding internal forces can be verified against the strength constraints. Considering the robustness and maturity of the finite element method (FEM) [44–46], this study employs FEM to compute the internal forces of structural members, thereby validating compliance with Equation (1)–Equation (11). The optimization process is then conducted using the PSO algorithm as illustrated in Fig 4.

## 4. Engineering application and implementation

### 4.1. Project background

The Phase I project of the Hangzhou Convention and Exhibition Center covers a total construction area of 643,200 m², consisting of eight exhibition halls. The basement is designed with reinforced concrete and steel columns, while the above-ground structure is a combination of a steel frame and a large-span truss steel roof. The steel structure includes six single-story standard exhibition halls and two two-story exhibition halls. Due to the large span of the exhibition hall trusses, which reach 81 meters, and the heavy lifting operations with large-section mechanical equipment, direct operation on the basement roof slab would inevitably cause cracking. To mitigate this risk, a heavy-load transfer steel platform, supported by the basement columns as proposed in this paper, is employed for the heavy lifting operations.

### 4.2. Design and optimization of the heavy-load transfer steel platform

**Fig 5:**
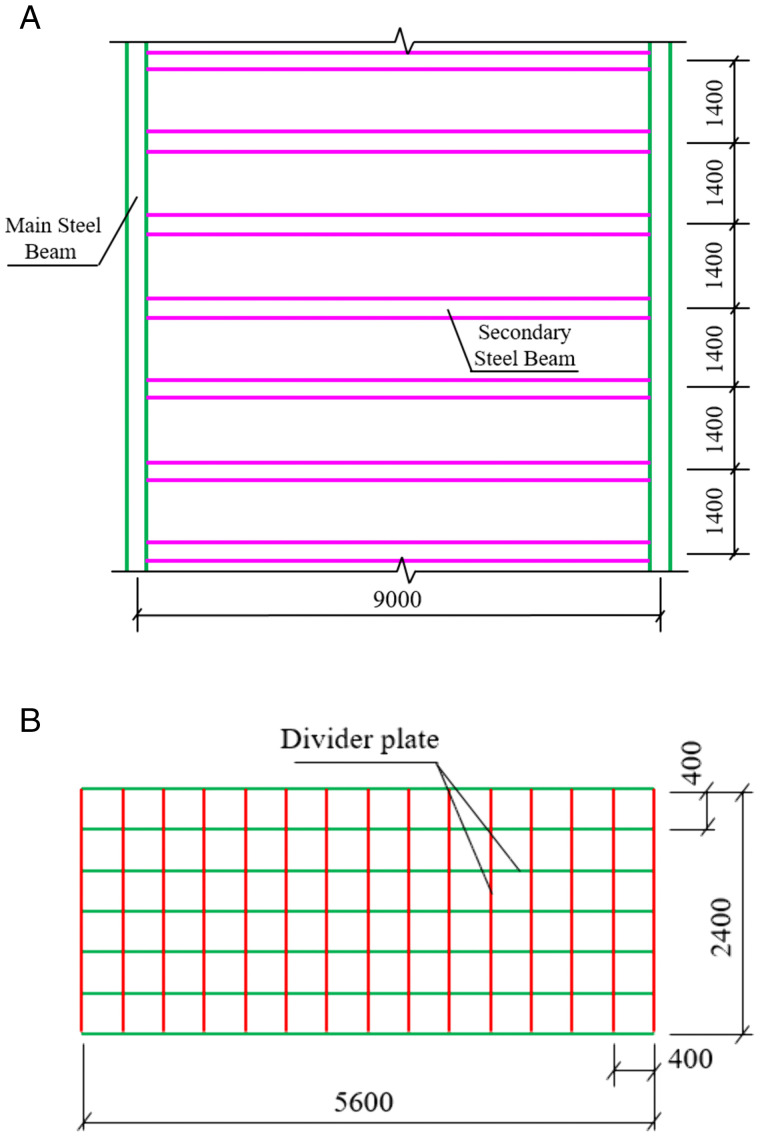
Structure of the heavy-load transfer steel platform: (a) Layout of primary and secondary beams; (b) Internal structure of the foundation box.

Based on the layout of the basement columns and lifting operation requirements, a heavy-load transfer steel platform was established, as shown in Fig 5. Considering that the internal force calculation of the components requires the finite element method, a corresponding finite element model needs to be established. The foundation box is modeled using four-node reduced integration shell elements to account for bi-directional bending and shear effects. The primary and secondary beams are modeled with linear beam elements. The basement columns within the influence area are also modeled using linear beam elements, with rigid end offsets considered at the column base plate and haunch areas. The platform-column connection is realized by setting reference points at the column center at the connection elevation and applying distributed coupling constraints to the shell/beam nodes around the base plate, ensuring the realistic transmission of force flow without restricting unnecessary rotations. The column base is treated as fixed at the foundation elevation to reflect the overall constraint of the column-pile-cap system during the lifting phase. The basement roof slab is not explicitly modeled, but is instead equivalently represented through geometric gaps and deformation checks to account for non-contact conditions, in line with the design intent. During mesh generation, the maximum element size does not exceed 1/10 of the minimum characteristic length to ensure computational accuracy. The calculation mainly considers the self-weight load and lifting load, with the lifting load applied as a surface force on the top plate of the foundation box, consistent with the contact area of the crane tracks/pads. A dynamic amplification factor of 1.20 is applied to account for the lifting load. The construction eccentricity is considered by offsetting the load surface to the most unfavorable position on site.

To verify the accuracy of the finite element analysis (FEA) model, field monitoring was conducted on the heavy-load transfer steel platform. Specifically, strain gauges were installed on the primary steel beams and foundation boxes to measure the stress distribution in the beams and the deflection of the foundation boxes during hoisting operations. The measured data were compared with the predicted results obtained from the FEA model, and a close correlation was observed between them. Under the maximum hoisting load, the deviation between the predicted and measured stress values of the steel beams was 5.2%, while the deviation between the predicted and measured maximum deflection of the foundation box was 3.7%. These findings indicate that the FEA model accurately captures the structural response of the platform under actual working conditions, providing a solid scientific basis for subsequent design optimization.

The initial components of the steel platform are listed in Table 2. Optimization of the platform was carried out using the design method established in this paper. The optimized components are also listed in Table 2. As can be seen from Table 2, the optimization primarily involves three key components: the foundation box, secondary beams, and primary beams.

**Table 2:** Comparison of steel platform specifications before and after optimization.

Component	Pre-Optimization Specification	Post-Optimization Specification	Cost Savings (%)
Foundation Box	Spacer PL200 × 20, spacing 400 mm	Spacer PL200 × 20, spacing 800 mm	44.68
Secondary Beam	B400 × 400 × 12 × 12	B200 × 200 × 10 × 10	58.33
Primary Beam	B400 × 400 × 20 × 20	B300 × 300 × 12 × 12	55.00

Before optimization, the foundation box used a spacer of PL200 × 20 with a spacing of 400 mm; after optimization, the spacing was adjusted to 800 mm while keeping the specifications unchanged. With consistent geometric parameters, the increased spacing reduces the number of spacers required in a given length of the foundation box by half, directly leading to a substantial reduction in material usage and processing procedures. The cost savings for the foundation box are 44.68%, meaning the engineering cost for this part is nearly halved while still meeting the required load-bearing capacity and stiffness.

The initial specifications for the secondary beam were B400 × 400 × 12 × 12, while after optimization, they were adjusted to B200 × 200 × 10 × 10. Clearly, the cross-sectional dimensions were reduced from 400 mm to 200 mm, and the thickness was also decreased, resulting in a significant reduction in steel consumption per unit length. The cost savings are 58.33%, with the optimized secondary beams costing less than half of the initial cost. Given the large number of secondary beams, this savings significantly impacts the overall project cost.

The primary beam initially had the specification B400 × 400 × 20 × 20, but after optimization, it was adjusted to B300 × 300 × 12 × 12. The cross-sectional size was reduced from 400 mm to 300 mm, and the thickness was decreased from 20 mm to 12 mm, resulting in a notable reduction in steel usage. The cost savings for the primary beam are 55.00%, which is comparable to the savings from the secondary beam.

**Fig 6:**
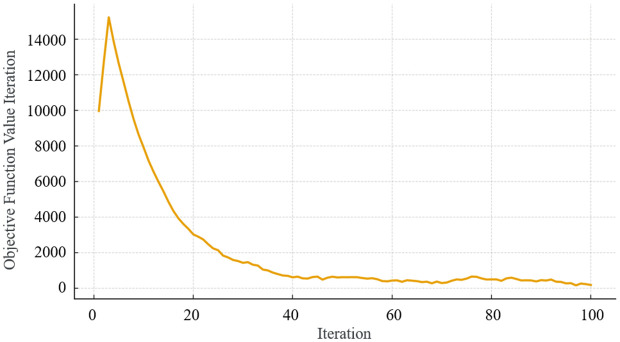
Iteration curve of the optimization process.

In order to further illustrate the efficiency and robustness of the Particle Swarm Optimization (PSO) in this specific engineering application, the convergence history curve of the PSO algorithm is plotted in Fig 6. As shown, the objective function value rapidly decreases in the initial iterations, and the algorithm reaches convergence after approximately 40 iterations, demonstrating both fast convergence speed and high stability. This confirms that the optimization process effectively searched the solution space and achieved a global optimal solution under the defined constraints.

**Fig 7:**
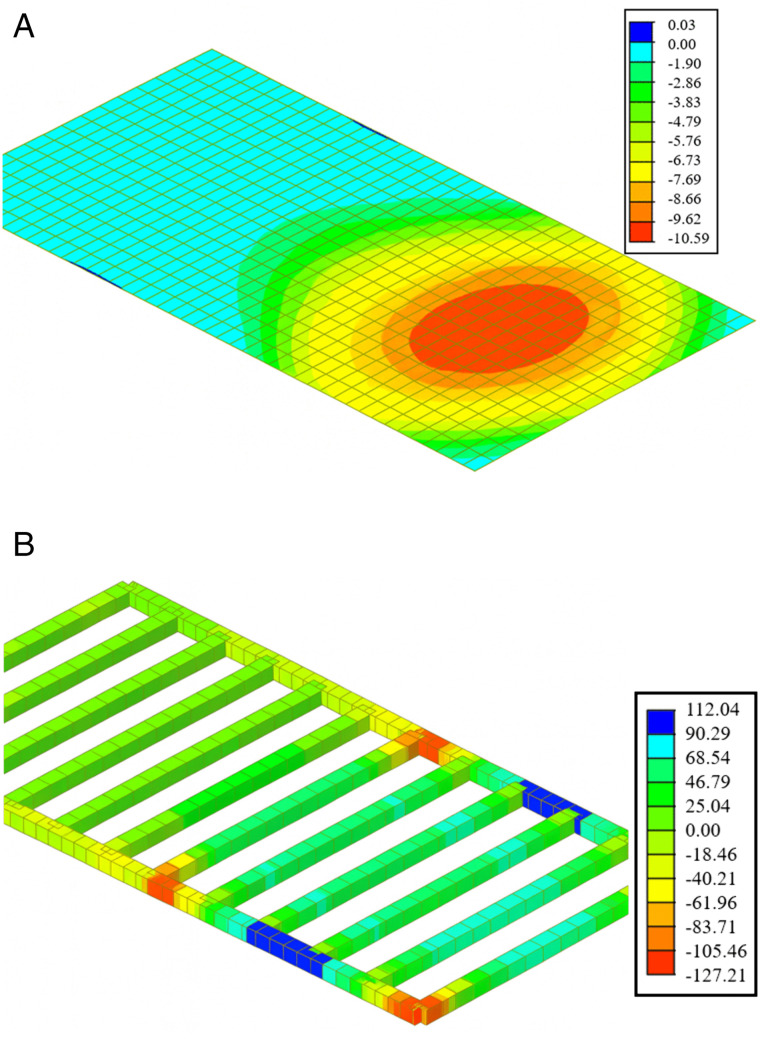
Optimized finite element analysis results of the heavy-load conversion steel platform: (a) Deflection (mm); (b) Stress (MPa).

The optimized finite element analysis results are shown in Fig 7. Fig 7(a) illustrates the finite element deflection distribution of the optimized heavy-load conversion steel platform. The deformation contour map indicates that the maximum vertical deflection occurs in the foundation box under load, with a peak value of 10.59 mm. The deflection gradually decreases in the surrounding areas, transitioning smoothly, suggesting that the overall stiffness of the platform is satisfactory and that the load is effectively distributed through the primary and secondary beam systems. Fig 7(b) presents the stress distribution of the steel beam under the same working conditions. The maximum equivalent stress occurs near the beam-column connection, with a peak value of 127.21 MPa, while most of the beam segments experience stress below 80 MPa. The calculation results show that both the deflection and stress of the platform meet the design limits for temporary hoisting structures, indicating that the optimized design has good strength, stiffness, and performance during the heavy-load hoisting construction phase.

**Fig 8:**
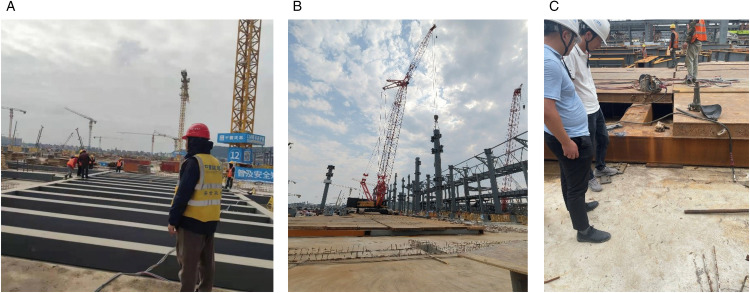
On-Site Construction of the Heavy-Load Transfer Steel Platform.

From the average savings across all three components—44.68% for the foundation box, 58.33% for the secondary beams, and 55.00% for the primary beams—the overall cost savings amounted to 52.67%. This optimization results in over a 50% reduction in material costs for the steel platform. In actual engineering applications, this savings is not only reflected in direct material procurement costs but also in the reduced transportation, installation, welding, and construction labor costs, leading to more significant overall economic benefits.

### 4.3. On-site construction of the heavy-load transfer steel platform

According to the design plan, the main components of the steel platform (such as the primary beams, secondary beams, and foundation boxes) were pre-fabricated in the factory and assembled on-site. As shown in Fig 8, the platform adopted a modular design, where all components were pre-fabricated in the factory for quick assembly on-site. During installation, the primary and secondary beams were first welded or bolted together to form a unified structure. All connection points underwent rigorous quality checks to ensure stability and avoid loosening.

Foundation boxes were then placed on top of the steel frame formed by the primary and secondary beams, ensuring that the lifting load was evenly distributed across the platform. Through reasonable placement of the foundation boxes, the load was effectively transmitted to the supporting system of the steel platform, rather than directly applying pressure to the basement roof slab. To avoid the load on the basement roof slab, the steel platform was designed to maintain a gap between the platform’s bottom and the roof slab, effectively isolating the heavy machinery load from the slab.

Once the steel platform assembly was complete, all installation joints underwent strict quality inspections, including non-destructive testing of welds and tensile tests on bolted connections. After the installation of each support point on the platform, load simulation tests were conducted to ensure that the platform could bear the design load.

### 4.4. Benefit analysis

The implementation of the heavy-load transfer steel platform effectively isolated the basement roof slab from the heavy lifting loads, preventing cracking or damage due to concentrated loads. The load was evenly distributed to the basement columns and foundation, avoiding structural damage caused by uneven load distribution in traditional scaffold reinforcement systems. This system effectively protected the safety of the underground structure and provided a stable foundation for subsequent construction work.

Compared to traditional scaffold reinforcement solutions, the installation process of the heavy-load transfer steel platform significantly shortened the construction cycle. The platform’s modular design enabled workers to rapidly assemble it on-site, saving a substantial amount of labor and time. According to the project’s cost evaluation, using the heavy-load transfer steel platform resulted in an approximately 12% reduction in overall project costs compared to using traditional scaffold reinforcement. Furthermore, since the steel platform and foundation boxes are reusable, the long-term material costs and resource consumption in subsequent construction phases were further reduced.

The heavy-load transfer steel platform is made from recyclable steel and foundation box materials, aligning with the requirements of green construction. Throughout the construction process, waste material recycling and resource reuse were effectively implemented, reducing the environmental impact of construction while aligning with current sustainability goals. The platform’s high durability and reusability significantly lower maintenance costs and extend the service life of the structure.

Compared to traditional scaffold reinforcement schemes, the use of an optimized heavy-load transfer steel platform can save approximately 12% in costs, with a corresponding 12% reduction in carbon emissions. When compared to the unoptimized heavy-load transfer steel platform, the optimized version reduces material waste in the foundation box, secondary beams, and main beams by 44.68%, 58.33%, and 55.00%, respectively. Corresponding carbon emissions are also reduced by 44.68%, 58.33%, and 55.00%. Additionally, the high durability and reusability of the heavy-load transfer platform further decrease maintenance costs and extend the service life of the structure.

In summary, the application of the heavy-load transfer steel platform successfully resolved the cracking issue caused by heavy lifting loads on the basement roof slab, while also improving construction safety and efficiency. Comparing it with traditional scaffold reinforcement, the platform demonstrates significant economic and technical advantages. In future large-scale underground structure construction projects, the heavy-load transfer steel platform will undoubtedly become an important reinforcement solution, warranting wider application in more projects.

### 4.5. Discussion

In this study, we proposed an innovative heavy-load transfer steel platform and evaluated its performance using a finite element analysis (FEA) model. Comparison with field measurement data confirmed the platform’s effective structural response and favorable mechanical performance during heavy lifting operations. However, despite the positive results, several factors remain that warrant further investigation and improvement.

Firstly, although the finite element analysis model successfully predicted the platform’s stress and deflection behavior, the model’s accuracy is still influenced by certain assumptions. For example, the model assumes uniformity in the platform’s structure and idealized boundary conditions, which may differ from actual engineering applications. Specifically, under irregular column grid layouts and complex geological conditions, the load transfer path of the platform may become more complicated, resulting in deviations between the actual performance and the model’s predictions. Therefore, future research should incorporate more complex real-world scenarios, including the effects of irregular column grid layouts and varying foundation types, in order to enhance the model’s applicability and accuracy.

Secondly, while this study utilized a Particle Swarm Optimization (PSO) algorithm for platform design optimization, the optimization process was primarily based on static loading conditions, without considering the dynamic load effects during the hoisting process. In actual construction, hoisting operations are often accompanied by sudden dynamic load variations, which could result in different structural responses compared to those predicted under static loads. Therefore, future studies should integrate dynamic analysis methods to assess the impact of transient dynamic loads on platform stability during hoisting operations, ensuring that the platform remains safe and reliable under more complex loading conditions.

Furthermore, the validation in this study mainly focused on the steel beams and foundation boxes of the platform, with insufficient experimental verification of other components, such as the connections between the foundation boxes and the basement columns. In practical applications, these connections may become critical points in the platform’s performance. Therefore, future research should place greater emphasis on monitoring and analyzing these connection areas to ensure the reliability of all structural joints, thereby improving the overall load-bearing capacity and stability of the platform.

Finally, although the proposed heavy-load transfer steel platform offers clear advantages in terms of reducing construction time, lowering material costs, and improving resource efficiency, its economic feasibility and applicability in certain cases, such as large-scale, multi-story, and complex underground structures, still require further investigation. For large-span or irregular buildings, custom designs of platform dimensions, materials, and connection methods may be required to meet the demands of various operating conditions. Consequently, future research should explore the application potential of the platform in complex engineering environments and develop more flexible and cost-effective design solutions.

In conclusion, while this study provides an effective solution in the form of the heavy-load transfer steel platform and validates its reliability and optimization through field measurements, several areas remain for further research. By incorporating more complex construction conditions and dynamic analysis methods, future studies will further advance the application and development of this technology, offering innovative solutions for the reinforcement of underground structures.

## 5. Conclusion

This study presents an innovative reinforcement solution for heavy lifting operations on basement roof slabs, which can otherwise lead to cracking due to concentrated lifting loads. The proposed heavy-load transfer steel platform, supported by the basement columns, effectively isolates the lifting load from the roof slab. Through a detailed analysis of the platform’s stress mechanisms, its design and optimization methods were established and applied to an actual engineering project. The feasibility and superiority of the proposed solution were verified, leading to the following conclusions:

(1) The innovative design of the heavy-load transfer steel platform effectively addresses the shortcomings of traditional scaffold reinforcement. Unlike traditional scaffolds, which suffer from uneven load distribution and are prone to cracking due to localized stress concentrations, the steel platform evenly distributes the load to the basement columns and ultimately to the foundation, effectively preventing roof slab cracking.
(2) Based on the stress analysis of various components of the steel platform, reasonable design standards were proposed, ensuring that the platform can bear lifting loads and maintain stability and safety under extreme load conditions.
(3) The optimized design of the steel platform significantly reduces engineering costs. By using an intelligent optimization particle swarm algorithm, the material usage was minimized, resulting in substantial cost savings in the foundation box, secondary beams, and primary beams, with an overall cost reduction of 52.67%. This optimization also reduced transportation, installation, and maintenance costs, providing significant economic benefits.
(4) In the Hangzhou Convention and Exhibition Center Phase I project, the heavy-load transfer steel platform successfully avoided cracking in the basement roof slab during lifting operations. The platform’s structure was stable, and its modular design significantly shortened the on-site installation time. It also allowed for repeated use in different lifting operations, reducing material costs and resource consumption in long-term construction, thus further improving the efficiency and economy of the project.

Although the platform demonstrates excellent engineering applicability, certain limitations still exist. When the spacing between basement columns is excessively large or the column grid layout is irregular, the load transfer path of the platform may become complex, thereby affecting its economic efficiency and construction convenience. In addition, the current method is mainly applicable to short-term temporary hoisting operations, and further research is required to investigate its performance evolution under long-term loading conditions. Future studies may focus on the following aspects: developing a more refined nonlinear finite element model to evaluate the platform’s safety reserve under complex loading conditions; integrating construction monitoring data to study deformation control behavior during different construction stages; and exploring new lightweight and high-strength materials to further reduce the platform’s self-weight and enhance its sustainability.

## Supporting information

S1 Data (TXT)
